# Association Between Dietary Niacin Intake and Life's Essential 8 Among US Adults (NHANES 2005–2018)

**DOI:** 10.1002/fsn3.70817

**Published:** 2025-09-02

**Authors:** Dan Wang, Fanli Yuan, Shouliang Hu

**Affiliations:** ^1^ Central Laboratory The First Affiliated Hospital of Yangtze University Jingzhou Hubei China; ^2^ Division of Nephrology The First Affiliated Hospital of Yangtze University Jingzhou Hubei China

**Keywords:** cardiovascular health, LE8, Life's Essential 8, NHANES, niacin

## Abstract

Limited evidence exists on the relationship between dietary niacin intake and Life's Essential 8 (LE8), a new metric for cardiovascular health. We analyzed data from 23,729 adults aged ≥ 20 years from the 2005–2018 National Health and Nutrition Examination Survey (NHANES). The LE8 score (range: 0–100) reflects health behaviors, health factors, and biomarkers. After adjusting for confounders, each 10‐unit increase in niacin intake was associated with 0.65‐point higher LE8 score (*β* = 0.65, 95% CI = 0.43–0.87; *p* < 0.001). Compared to the lowest tertile, the highest tertile of niacin intake had a 1.83‐point higher LE8 score (*β* = 1.83, 95% CI = 1.02–2.64; *p* < 0.001). Restricted cubic spline analysis indicated a linear dose–response relationship (P‐nonlinearity = 0.578). Stratified analyses confirmed significant effect modification, with niacin‐LE8 associations most pronounced in geriatric strata (*β* = 1.91, 95% CI = 1.08–2.74; P‐interaction = 0.002), amplified among non‐Hispanic Black (*β* = 1.27, 95% CI = 0.64–1.91) and White (*β* = 1.20, 95% CI = 0.77–1.64) populations versus attenuated effects in Mexican Americans (*p* = 0.003), enhanced across socioeconomic gradients (highest PIR tertile: *β* = 1.08, 95% CI = 0.56–1.60; *p* = 0.025), and exhibiting 86% magnitude elevation in cardiovascular comorbidity subgroups (*β* = 1.69, 95% CI = 0.59–2.79; *p* = 0.023). While these cross‐sectional findings suggest a beneficial relationship between dietary niacin intake and cardiovascular health, causality cannot be confirmed and residual confounding may remain. These findings may have implications for dietary guidance aimed at cardiovascular health at the population level.

## Graphical abstract

Our analysis of NHANES data (2005–2018) shows a positive association between dietary niacin intake and LE8 scores, with stronger effects in non‐Hispanic Whites and Blacks compared to Mexican Americans. The association is also more pronounced in younger and older adults than in middle‐aged individuals. These findings suggest that niacin intake might be associated with improved cardiovascular health, with varying benefits across demographic groups.

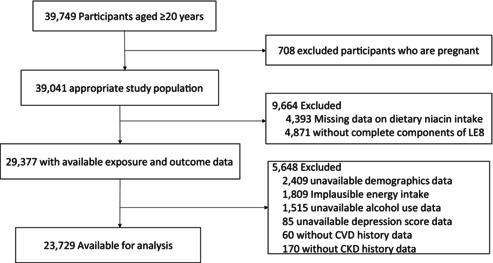

## 1 Introduction

While cardiovascular disease (CVD) mortality has substantially decreased over the past few decades, it remains a prominent public health issue in both the United States and Western Europe (Liu et al. 2023; Sidney et al. 2016). In response, the American Heart Association (AHA) recently introduced Life's Essential 8 (LE8), a framework that defines eight critical components for maintaining optimal cardiovascular health: blood glucose, blood pressure, blood lipids, exposure to nicotine, sleep duration, physical activity, diet, and body mass index (Lloyd‐Jones, Allen, et al. 2022). This comprehensive framework emphasizes the importance of addressing modifiable behaviors and clinical indicators to improve population‐wide cardiovascular health.

Dietary factors play a central role in cardiovascular outcomes, with various nutrients having distinct effects (An et al. 2022; Fang et al. 2023; Mente et al. 2023). Niacin (vitamin B3), in particular, has long been recognized for its lipid‐modifying properties, and its role in metabolic and cardiovascular health has been widely studied (Jenkins et al. 2021; Zhang et al. 2021). Despite the established role of niacin in lipid metabolism, its relationship with the newly introduced LE8 metric has not been comprehensively examined. This gap is important, as LE8 reflects a more holistic assessment of cardiovascular health beyond lipid levels alone.

Although some clinical studies have suggested that niacin supplementation may provide cardiovascular benefits (Bruckert et al. 2010; Pan et al. 2024; Siniawski et al. 2016; Song et al. 2023; Taylor et al. 2009), others have reported limited efficacy or raised concerns regarding potential adverse effects (Ferrell et al. 2024; Garg et al. 2017; Lim 2024). These findings are often based on specific clinical populations, such as individuals with hyperlipidemia, and do not capture the broader picture of cardiovascular health in the general population. There remains a lack of population‐level studies exploring how dietary niacin intake relates to overall cardiovascular health as quantified by LE8.

To address this gap, we investigated the association between dietary niacin intake and LE8 scores in a large, nationally representative sample of U.S. adults. We also examined how this relationship may vary across racial and age subgroups and evaluated the influence of demographic, socioeconomic, and lifestyle factors. By examining niacin intake in the context of the LE8 framework, this study provides novel insights that may inform more targeted dietary guidance and public health strategies for reducing CVD burden in diverse populations.

## 2 Methods

### 2.1 Study Population

In NHANES, a series of continuous, multistage, stratified, and nationally representative surveys of the U.S. civilian noninstitutionalized population are conducted. A detailed description of the NHANES survey methods and analytic guidelines is available (Preedy and Watson 2010). To gather rich data on health topics, such as demographics, socioeconomics, diets, and health‐related issues, NHANES conducted home interviews, followed by a mobile examination center where blood samples were taken. Consent from all participants was secured through NHANES (Preedy and Watson 2010).

**FIGURE 1:**
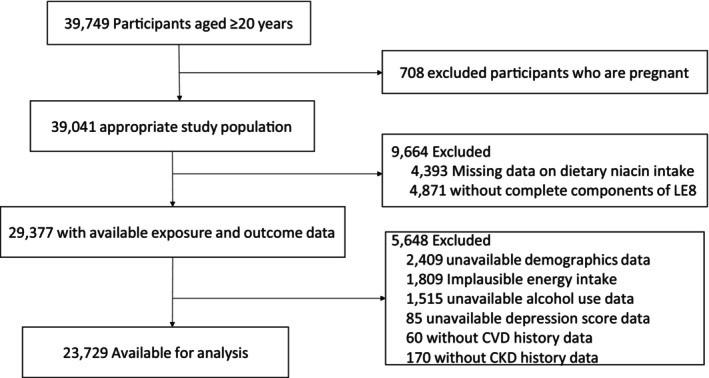
Flowchart of study population selection from NHANES 2005–2018.

23,729 adult participants in this cross‐sectional study were screened from NHANES 2005–2018. The study procedure is illustrated in Figure 1 (Study Flowchart). Those satisfying any of the predetermined principles were ruled out from the research: age under 20 years, pregnancy, incomplete data on dietary niacin intake or LE8 components, missing covariate information, or implausible total energy intake. These exclusions follow standard NHANES analytic protocols and prior research practices to ensure data quality and consistency (Li et al. 2025; Yang et al. 2024). Thresholds for energy intake for men, intake below 800 kcal or above 4200 kcal per day are considered extreme. Similarly, for women, an intake of less than 600 or more than 3500 kcal per day is considered extreme (Satija et al. 2017).

### 2.2 Dietary Data

An Automated Multiple‐Pass Method (AMPM) was used to conduct the dietary interviews as part of What We Eat in America. Various studies have explored the AMPM method's dependability, which was developed to ensure an accurate approach for gathering dietary intake data for extensive national surveys (Blanton et al. 2006; Rhodes et al. 2013; Rumpler et al. 2008). In the NHANES study, participants underwent two interviews for 24‐h dietary recall, where they were prompted to remember the kinds and amounts of food they had eaten in the 24 h leading up to the interview. Initial dietary recalls were made through personal interviews at the Mobile Examination Centre (MEC), whereas subsequent recalls took place via telephone around three to ten days afterward. The Nutrient Database and USDA Dietary Research Food were used to calculate the nutrient content and all foods' food composition consumed (Blanton et al. 2006; Rhodes et al. 2013). This study used participants' first day's meal recall value as the main analysis and used two days' intake as the sensitivity analysis. This approach is widely used in NHANES‐based nutritional epidemiology (Pan et al. 2024; Yang et al. 2024).

### 2.3 Definition of LE8

The Life's Essential 8 (LE8), comprises eight key components of cardiovascular health (CVH), organized into two primary categories. It was recently created by the American Heart Association (AHA). The first category, behavioral, encompasses aspects such as dietary habits, sleep quality, physical exercise, and tobacco use.

The second category, biomedical, includes body mass (assessed by body mass index [BMI], which is figured as weight split using height's square in kilograms per square meters), blood pressure, lipid (non‐HDL cholesterol), and blood sugar (measured through fasting glucose or hemoglobin A1c, alongside the presence of diabetes). Additionally, a subset of this domain focuses on blood biomarkers, specifically lipid and glycemic indices. The methods used to assess and quantify each LE8 component are provided in Table S1 (LE8 Component Scoring Criteria) (Lloyd‐Jones, Allen, et al. 2022). The general LE8 score was computed by us, along with individual scores for each subdomain and each domain, measured on an extent from 0 to 100 points. Higher values reflect better cardiovascular health. Scores for the overall, blood biomarker, biomedical, and behavioral domains were computed by averaging the individual component scores. Based on these scores, cardiovascular health is classified as low (0–49), moderate (50–79), or high (80–100).

### 2.4 Assessment of Covariates

Race and ethnicity were determined based on participant‐reported data from the NHANES, with four categories: non‐Hispanic Black, non‐Hispanic White, Mexican American, and other (which included other individuals identifying with multiple non‐Hispanic races, other Hispanic, and other non‐Hispanic). Age was stratified into three categories: 65 years and above, 40 to 65 years, and 20 to 40 years. These categories were included as potential confounders. Educational attainment was categorized into three tiers: some college or higher education, a diploma from high school, and an education level below high school.

The PIRs of families were segmented into three categories: greater than 3.0, between 1.0 and 3.0, and below 1.0. Smoking status was classified into current smokers (100 or more cigarettes), former smokers (100 or more cigarettes but had quit), and never smokers (individuals who have smoked fewer than 100 cigarettes throughout their entire lifetime). Alcohol consumption was self‐reported and divided into three groups: current drinkers (alcohol consumed within the past year), former drinkers (12 or more drinks consumed in a lifetime but none in the past year), and never drinkers (fewer than 12 alcoholic drinks consumed in a lifetime). Current drinkers included those who reported any level of alcohol consumption, ranging from occasional to daily intake, regardless of the quantity consumed. A participant is considered physically active if he or she engages in moderate to vigorous physical activity for no less than 150 min per week, following the guidelines provided by the Centers for Disease Control and Prevention (CDCP). Those not meeting this threshold were considered inactive. BMI was classified into three ranges: ≥ 30.0, between 29.9 and 25.0, and < 25.0. Data regarding dietary supplement usage over the past 30 days have been systematically collected by trained personnel. Dyslipidemia was defined by meeting one or more of the following criteria: below 40 mg/dL HDL, 130 mg/dL LDL or higher, 150 mg/dL or more triglycerides (to translate the value to millimole per liter, multiply it using the factor 0.0113), or ≧ 200 mg/dL total cholesterol (to convert, multiply by 0.0259) (Lin et al. 2017). Clinical history included self‐reported diagnoses of cardiovascular disease (CVD) and chronic kidney disease (CKD), as well as use of lipid‐lowering medications and antihypertensive medications, both of which were included as additional covariates to account for treatment‐related confounding. Furthermore, total energy intake (kcal/day) was treated as a continuous covariate to account for overall dietary quantity and reduce the risk of residual confounding in the relationship between niacin intake and cardiovascular health. Evaluated using the Healthy Eating Index 2015 (HEI‐2015), the quality of diet is based on the patterns recommended in the Dietary Guidelines for Americans, 2015–2020 (Hauk 2016) and 2020–2025 (Thompson 2021). The scoring system for the HEI‐2015 ranges from 0 to 100, detailed in Table S2 (HEI‐2015 Components and Scoring Standards), with higher grades reflecting a healthfuller diet.

### 2.5 Statistical Analysis

Considering NHANES's intricate sampling structure, this research integrates primary sampling units, strata, and sample weights into all analyses. In the main analysis, we used the Day 1 dietary sample weights (WTDRD1), which are specifically recommended when analyzing dietary recall data from Day 1 alone or in conjunction with the Mobile Examination Center (MEC) data. These weights were divided by the number of NHANES cycles to account for the combined survey design. Because our study combined data across seven 2‐year cycles from 2005 to 2018, we constructed a 14‐year analytic weight by dividing WTDRD1 by 7, following NHANES analytic guidance. This adjustment ensures appropriate representation across the pooled dataset. The procedure is consistent with the NHANES recommendation for multi‐cycle analyses: “New multi‐year sample weights can be computed by simply dividing the two‐year sample weights by the number of two‐year cycles in the analysis.” (CDC/NCHS Weighting Tutorial, https://wwwn.cdc.gov/nchs/nhanes/tutorials/Weighting.aspx).

In the sensitivity analysis that included only participants with complete two‐day dietary recalls, we applied the two‐day dietary weights (WTDR2D), also adjusted by dividing by the number of cycles, as recommended by NHANES for analyses using both Day 1 and Day 2 data (https://wwwn.cdc.gov/Nchs/Data/Nhanes/Public/2009/DataFiles/DR1IFF_F.htm).

For categorical variables, consequences are reported as corresponding unweighted frequencies and weighted percentages. Normally distributed continuous variables are expressed as standard deviation and mean (SD), and non‐normally distributed continuous variables are expressed as interquartile range and median (IQR).

Variables' normality was estimated through the Kolmogorov–Smirnov test, Q‐Q plots, or histogram distributions. The chi‐square test or Fisher's exact test was used to evaluate between‐group differences in categorical variables. The Kruskal‐Wallis H test and One‐Way ANOVA are for non‐normally distributed data. For multiple comparisons, either the Tukey or LSD method was applied.

The influence of dietary niacin intake on LE8 scores was assessed using weighted linear regression models, which provided regression coefficients (β) along with 95% confidence intervals (CI), adjusting for key covariates. Niacin intake was evaluated both as a continuous variable, where β indicates a 10‐point increase and as a categorical variable based on tertiles.

In Model 1, no adjustments were built for covariates. In contrast, adjustments were included by Model 2 for racial/ethnic background, gender, and age. Additionally, further adjustments were involved by Model 3, including education level, marital status, PIR, history of CVD and CKD, drinking status, and depression. The assignment of median values to each category is for the examination of linear trends. Niacin intake was also categorized into tertiles to explore trends and potential nonlinear associations, with the *p*‐value for trend calculated.

To investigate the nonlinear dose–response relationships between LE8 scores and niacin consumption, restricted cubic spline models were utilized, incorporating four nodes situated at the 5th, 35th, 65th, and 95th percentiles. Nonlinear assessment included quadratic terms' inclusion in the regression model. Based on the resulting smoothed curves, a two‐part linear regression model was formulated to detect potential threshold effects while adjusting for potential confounders.

Interaction and subgroup analyses were carried out, and the likelihood proportion examination was utilized to evaluate the interaction effects across subgroups. Additionally, multiple sensitivity analyses were performed to evaluate the results' robustness and to examine different association models' effects. *p*‐values and effect sizes from all models were compared and documented.

All statistical analyses were performed by applying R Statistical Software in conjunction with the FreeStatistics analysis platform. FreeStatistics is an intuitive software designed for easy data analysis and visualization, powered by R with a Python‐based graphical user interface (GUI) (http://www.clinicalscientists.cn/freestatistics, Version 1.9.2, Beijing, China). It facilitates reproducible research and interactive computations, requiring minimal effort for most statistical operations. A bilateral *p*‐value below 0.05 was deemed statistically to be significant.

Throughout this manuscript's preparation, ChatGPT was utilized to improve the clarity and readability of the text. After utilizing this tool, the authors conducted a thorough review and made revisions where needed, taking full responsibility for the accuracy and integrity of the final published work.

## 3 Results

### 3.1 Characteristics of Participants

Baseline characteristics of the participants categorized by tertiles of dietary niacin intake are summarized in Table 1 (Baseline Characteristics by Dietary Niacin Intake Level). This study encompassed 23,729 participants, with a mean age of 49.46 years (standard deviation [SD] 16.83). Among these participants, 5558 individuals were aged 65 years or older, representing 18.10% of the cohort. The gender distribution included 11,828 men, accounting for 48.85% (weighted), and 11,901 women, making up 51.15% (weighted) of the total sample. A total of 3518 participants (7.85%) were identified as Mexican American, while 4607 individuals (9.51%) were classified as non‐Hispanic Black. The majority of the cohort consisted of 11,043 participants (70.35%) who identified as non‐Hispanic White and 4561 individuals (12.29%) fell into the “other” category. The average score for Life's Essential 8 among these groups was 64.42, with a standard deviation of 14.88. Specifically, tertile 1 comprised 7909 participants whose intake was below 17.7 mg per day; 7910 participants in tertile 2 [17.7–27.1 mg/day]; and 7910 participants in tertile 3 [≥ 27.1 mg/day] are summarized. Participants with higher dietary niacin consumption exhibited distinct demographic and lifestyle characteristics compared to those with the lowest niacin consumption. Individuals with elevated niacin consumption tended to be predominantly male and younger. They demonstrated higher educational attainment and a greater proportion reported higher family income levels. A larger percentage identified as Non‐Hispanic White and a higher proportion were married or cohabiting. Furthermore, rates of alcohol consumption were notably higher among those with higher dietary niacin intake. Interestingly, individuals with elevated niacin intake had a lower prevalence of cardiovascular and renal disease history, as well as a lower incidence of depressive state.

**TABLE 1:** Baseline characteristics of participants by tertiles of dietary niacin intake.

Characteristic	Total (*N* = 23,729)	Tertile 1 (Niacin intake < 17.7 mg/day) *N* = 7909	Tertile 2 (17.7–27.1 mg/day) *N* = 7910	Tertile 3 (> 27.1 mg/day) *N* = 7910	*p*
Age, mean (SD), years	49.46 (16.83)	51.89 (17.57)	50.19 (16.64)	46.29 (15.98)	**< 0.0001**
Age, *n* (%)					
< 40	8017.00 (36.47)	2334.00 (32.79)	2545.00 (33.68)	3138.00 (42.22)	**< 0.0001**
40–65	10,154.00 (45.43)	3271.00 (43.78)	3466.00 (47.80)	3417.00 (44.58)	
≥ 65	5558.00 (18.10)	2304.00 (23.43)	1899.00 (18.52)	1355.00 (13.20)	
Sex, *n* (%)					
Male	11,828.00 (48.85)	2571.00 (28.12)	3790.00 (45.74)	5467.00 (69.28)	**< 0.0001**
Female	11,901.00 (51.15)	5338.00 (71.88)	4120.00 (54.26)	2443.00 (30.72)	
Race/ethnicity, *n* (%)					
Mexican American	3518.00 (7.85)	1203.00 (8.00)	1163.00 (7.77)	1152.00 (7.81)	**< 0.0001**
Non‐Hispanic black	4607.00 (9.51)	1680.00 (11.24)	1467.00 (8.80)	1460.00 (8.71)	
Non‐Hispanic white	11,043.00 (70.35)	3466.00 (67.48)	3756.00 (71.50)	3821.00 (71.67)	
Others	4561.00 (12.29)	1560.00 (13.28)	1524.00 (11.93)	1477.00 (11.81)	
Marital status, *n* (%)					
Married or living with a partner	14,400.00 (63.47)	4429.00 (58.69)	4922.00 (64.95)	5049.00 (66.12)	**< 0.0001**
Living alone	9329.00 (36.53)	3480.00 (41.31)	2988.00 (35.05)	2861.00 (33.88)	
Education level, *n* (%)					
Less than high school	5328.00 (14.07)	2098.00 (17.66)	1739.00 (13.27)	1491.00 (11.80)	**< 0.0001**
High school or equivalent	5432.00 (23.06)	1881.00 (24.83)	1764.00 (22.08)	1787.00 (22.50)	
Above high school	12,969.00 (62.87)	3930.00 (57.51)	4407.00 (64.65)	4632.00 (65.70)	
PIR, mean (SD)	2.59 (1.63)	2.39 (1.59)	2.62 (1.64)	2.75 (1.65)	**< 0.0001**
Drinking status, *n* (%)					
Never	6989.00 (27.64)	2676.00 (31.64)	2353.00 (28.02)	1960.00 (23.91)	**< 0.0001**
Former	3791.00 (12.90)	1500.00 (15.29)	1246.00 (12.99)	1045.00 (10.79)	
Current	12,949.00 (59.46)	3733.00 (53.07)	4311.00 (58.99)	4905.00 (65.30)	
Depression, *n* (%)					
No	21,890.00 (92.84)	7137.00 (90.73)	7335.00 (93.31)	7418.00 (94.16)	**< 0.0001**
Yes	1839.00 (7.16)	772.00 (9.27)	575.00 (6.69)	492.00 (5.84)	
CVD history, *n* (%)					
No	21,215.00 (91.75)	6879.00 (89.70)	7051.00 (91.81)	7285.00 (93.43)	**< 0.0001**
Yes	2514.00 (8.25)	1030.00 (10.30)	859.00 (8.19)	625.00 (6.57)	
CKD history, *n* (%)					
No	19,335.00 (85.70)	6126.00 (81.84)	6440.00 (85.87)	6769.00 (88.79)	**< 0.0001**
Yes	4394.00 (14.30)	1783.00 (18.16)	1470.00 (14.13)	1141.00 (11.21)	
Life's Essential 8 scores, mean (SD)	64.42 (14.88)	62.83 (15.23)	64.50 (14.82)	65.94 (14.43)	**< 0.0001**
Behavioral domain^a^, mean (SD)	62.83 (19.73)	60.85 (20.22)	63.07 (19.54)	64.57 (19.25)	**< 0.0001**
Diet	40.20 (31.38)	38.56 (31.66)	40.69 (31.31)	41.34 (31.10)	**< 0.0001**
Smoking status	66.77 (39.71)	68.05 (39.64)	67.44 (39.10)	64.83 (40.31)	**< 0.0001**
Physical activity	62.59 (44.95)	56.61 (46.17)	62.05 (45.07)	69.11 (42.69)	**< 0.0001**
Sleep health	81.76 (25.20)	80.17 (26.31)	82.10 (25.15)	83.00 (24.00)	**< 0.0001**
Biomedical domain^b^, mean (SD)	66.01 (19.98)	64.80 (20.23)	65.92 (19.83)	67.32 (19.78)	**< 0.0001**
Body mass index	60.68 (33.42)	59.93 (33.82)	60.58 (33.39)	61.52 (33.03)	**0.0104**
Blood pressure	57.81 (35.15)	56.19 (36.08)	57.82 (35.37)	59.42 (33.91)	**< 0.0001**
Glycemia	81.26 (26.30)	79.46 (27.04)	80.63 (26.49)	83.68 (25.16)	**< 0.0001**
Blood lipids	64.31 (31.45)	63.63 (31.65)	64.66 (31.18)	64.65 (31.51)	0.0611
Blood biomarkers subdomain^c^, mean (SD)	72.78 (21.85)	71.54 (22.34)	72.64 (21.74)	74.17 (21.39)	**< 0.0001**
Energy, mean (SD), kcal	2032.97 (758.63)	1487.52 (505.37)	2014.87 (597.03)	2596.45 (706.70)	**< 0.001**
Lipid‐lowering drugs, *n* (%)					
No	19,223.00 (83.00)	6269.00 (82.00)	6323.00 (82.00)	6631.00 (84.70)	**< 0.001**
Yes	4506.00 (17.00)	1640.00 (18.00)	1587.00 (18.00)	1279.00 (15.30)	
Antihypertensive drugs, *n* (%)					
No	16,234.00 (72.90)	5074.00 (69.00)	5316.00 (71.90)	5844.00 (77.10)	**< 0.001**
Yes	7495.00 (27.10)	2835.00 (31.00)	2594.00 (28.10)	2066.00 (22.90)	

*Note:* Values are presented as unweighted counts with weighted percentages in parentheses, with percentages calculated using population‐weighted survey design adjustments. Bold *p*‐values signify values less than the significance level of 0.05.

Abbreviations: CKD, chronic kidney disease; CVD, cardiovascular disease; NHANES, National Health and Nutrition Examination Survey; PIR, poverty‐to‐income ratio.

^a^ Behavioral domain includes diet, physical activity, smoking status, and sleep duration.

^b^ Biomedical domain includes body mass index, blood pressure, blood lipids (non–high‐density lipoprotein cholesterol), and glycemia.

^c^ Blood biomarkers subdomain includes blood lipids and glycemia.

### 3.2 Dietary Niacin Intake and LE8 Scores

**FIGURE 2:**
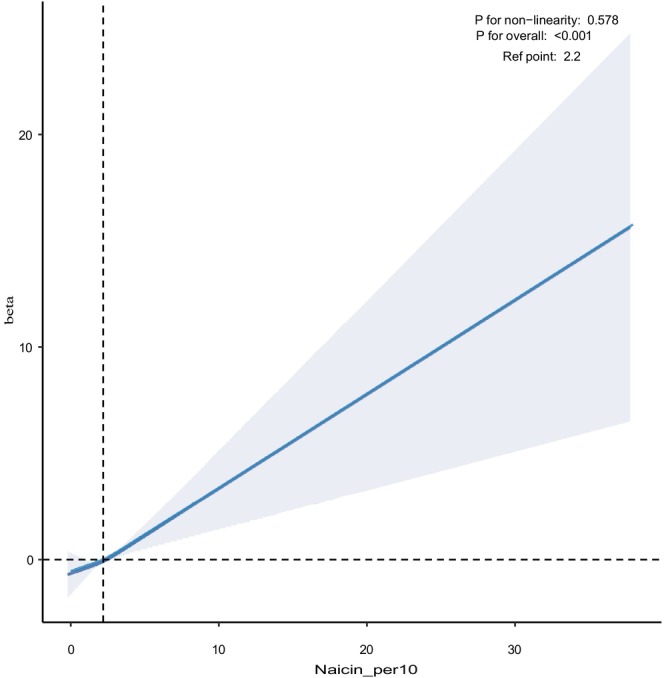
Restricted cubic spline showing dose–response between dietary niacin intake and LE8 scores.

The adjusted smoothed plots suggest a linear correlation between dietary niacin consumption and the LE8 scores in Figure 2 (*p* for overall < 0.001; P for nonlinearity = 0.578, with the highest and lowest 0.5% of dietary niacin consumption levels trimmed). As dietary niacin consumption increased, the LE8 scores exhibited an upward trend.

The results of univariate analyses of LE8 scores by participant characteristics are available in Table S3 (Univariate Analyses of LE8 Scores). Univariate regression analyses identified significant associations between Life's Essential 8 (LE8) scores and multiple demographic and clinical variables, including racial background, sex, age category, marital status, alcohol consumption patterns, depressive symptoms, educational attainment, poverty‐income ratio, total caloric intake, use of antihypertensive medications, utilization of lipid‐lowering agents, cardiovascular disease history, and chronic kidney disease status.

Results from multivariate regression models assessing the association between dietary niacin intake and LE8 scores are shown in Table 2 (Multivariable Associations between Niacin Intake and LE8 Scores). In the multivariate linear regression model modified for potential confounders, a 10‐unit increment in dietary niacin intake was associated with a 0.65‐point (*β* = 0.65, 95% CI = 0.43–0.87; *p* < 0.001) higher LE8 scores (Table 2, Model 3). A 0.65‐point increase in the LE8 score is associated with a modest but potentially meaningful improvement in cardiovascular health expectancy. LE8 is a composite metric that estimates the number of years an individual is expected to live in good cardiovascular health across eight modifiable lifestyle domains, including diet, physical activity, smoking, and sleep. Even small increases in LE8 may be associated with cumulative improvements in these health behaviors and a reduction in long‐term risk of chronic diseases. Compared with the lowest tertile of dietary niacin intake, the multivariable‐adjusted regression coefficients (95% CIs) for the LE8 scores were 0.59 (*β* = 0.59, 95% CI = −0.10 to 1.29; *p* = 0.094) for the second tertile and 1.83 (*β* = 1.83, 95% CI = 1.02–2.64; *p* < 0.001) for the highest tertile of dietary niacin intake (Table 2, Model 3). Subdomain analyses of LE8 components by niacin intake are presented in Table S4 (Associations with LE8 Subdomain Scores). After adjusting for possible confounders, a 10‐unit increment in dietary niacin consumption was significantly related to a 1.01‐point (*β* = 1.01, 95% CI = 0.69–1.33; *p* < 0.001) higher health behaviors domain score, a non‐significant 0.28‐point (*β* = 0.28, 95% CI = −0.01 to 0.58; *p* = 0.059) higher health factors domain score, and a 0.44‐point (*β* = 0.44, 95% CI = 0.10–0.77; *p* = 0.012) higher blood biomarkers domain score, respectively (Table S4).

**TABLE 2:** Multivariable associations between day 1 dietary niacin intake and Life's Essential 8 scores.

Variable	*β* ^e^ (95% CI)						
	No.	Model 1^a^	*p*	Model 2^b^	*p*	Model 3^c^	*p*
Dietary niacin intake	23,729	**0.77 (0.56, 0.97)**	**< 0.001**	**0.62 (0.42, 0.82)**	**< 0.001**	**0.65 (0.43, 0.87)**	**< 0.001**
Tertiles							
Q1 (< 17.7 mg/day)	7909	Ref^d^		Ref^d^		Ref^d^	
Q2 (17.7–27.1 mg/day)	7910	**1.09 (0.33, 1.86)**	**0.006**	**1.12 (0.38, 1.86)**	**0.003**	0.59 (−0.10, 1.29)	0.094
Q3 (≥ 27.1 mg/day)	7910	**2.43 (1.69, 3.16)**	**< 0.001**	**2.18 (1.44, 2.91)**	**< 0.001**	**1.83 (1.02, 2.64)**	**< 0.001**
*P* for trend			**< 0.001**		**< 0.001**		**< 0.001**

^a^ Model1: Crude.

^b^ Model2: Age/sex/race‐adjusted.

^c^ Model3: Fully adjusted.

^d^ Ref: Reference.

^e^ *β* coefficients represent the change in Life's Essential 8 scores per 10 mg/day increase in dietary niacin intake. Bold *p*‐values signify values less than the significance level of 0.05. Bold confidence intervals indicate intervals that do not include the null value for that estimate (0 for β coefficients).

### 3.3 Stratified and Sensitivity Analyses

**FIGURE 3:**
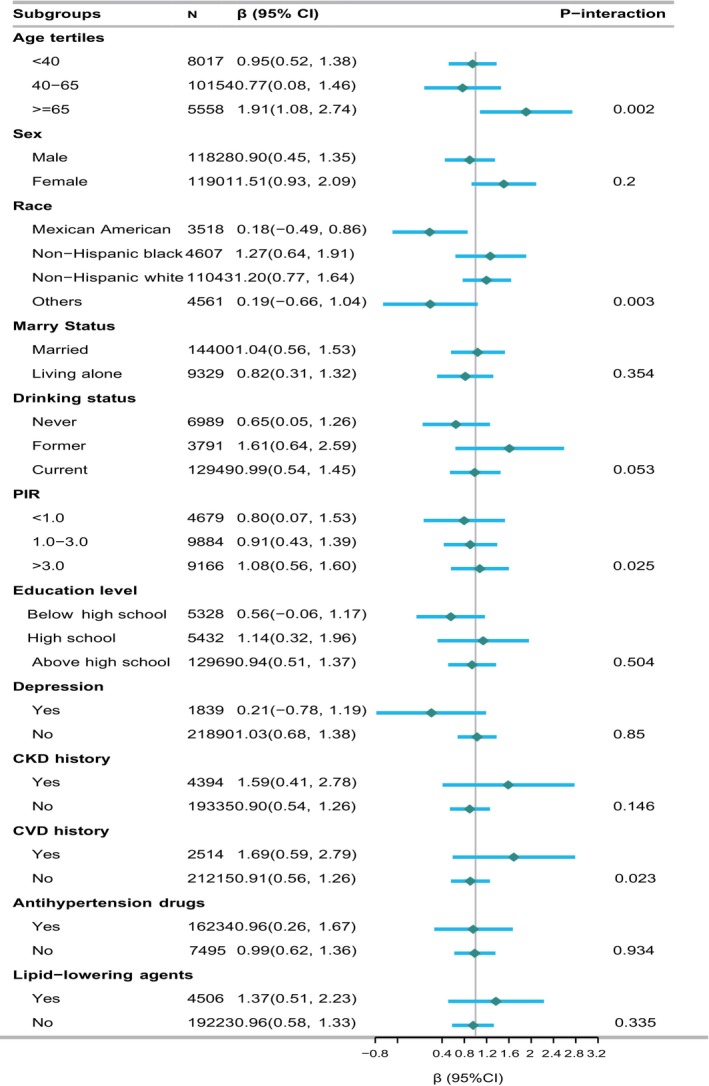
Subgroup analyses of the association between dietary niacin intake and LE8 scores across demographic and clinical characteristics.

Stratified analysis by age tertiles, sex, race/ethnicity, marital status, alcohol consumption, poverty income ratio (PIR), education level, depression status, and history of chronic kidney disease (CKD) and cardiovascular disease (CVD) is illustrated in Figure 3 (Subgroup Analyses of Niacin and LE8 Scores). By race/ethnicity, stronger positive associations were identified among non‐Hispanic White (*β* = 1.20, 95% CI = 0.77–1.64; *p* < 0.001) and non‐Hispanic Black participants (*β* = 1.27, 95% CI = 0.64–1.91; *p* < 0.001), whereas the relationship was not statistically significant among Mexican Americans (*β* = 0.18, 95% CI = −0.49 to 0.86; *p* = 0.59), with a significant interaction across racial groups (P‐interaction = 0.003). Age‐stratified analyses also revealed a nonlinear pattern, with greater benefits observed in younger adults (< 40 years: *β* = 0.95, 95% CI = 0.52–1.38; *p* < 0.001) and older individuals (≥ 65 years: *β* = 1.91, 95% CI = 1.08–2.74; *p* < 0.001) compared to those aged 40–65 years (*β* = 0.77, 95% CI = 0.08–1.46; *p* = 0.03; P‐interaction = 0.002). Income level also modified the association, with the highest effect estimate found in individuals with a poverty‐income ratio (PIR) > 3.0 (*β* = 1.08, 95% CI = 0.56–1.60), in contrast to weaker associations in lower‐income groups (P‐interaction = 0.025). Additionally, a more pronounced association was noted among participants with a history of cardiovascular disease (CVD) (*β* = 1.69, 95% CI = 0.59–2.79) relative to those without CVD (*β* = 0.91, 95% CI = 0.56–1.26; P‐interaction = 0.023). No significant interaction was detected for sex, marital status, education level, depressive symptoms, alcohol consumption, or CKD history (Figure 3). Sensitivity analysis using the two days' intake is shown in Table S5 (Sensitivity Analysis Using Two‐Day Niacin Intake data). In sensitivity analyses, we used both day 1 and day 2 dietary niacin data, and the results of the primary analyses were similar to the present results (Table S5).

## 4 Discussion

Recent updates to the American Heart Association's (AHA's) cardiovascular health assessment framework, LE8, have been introduced (Lloyd‐Jones, Allen, et al. 2022). This model expands upon and refines the original metrics of Life's Simple 7 (Lloyd‐Jones et al. 2010) by incorporating eight key components. These components are divided into two categories: health behaviors (physical activity, diet, sleep, nicotine exposure) and health factors (blood pressure, blood lipids, blood glucose, and body mass index). While the primary purpose of LE8 is to measure and track CVH over time, its utility extends to predicting future cardiovascular disease (CVD) risk and other health outcomes throughout the lifespan. This predictive capability is particularly valuable for younger populations, for whom traditional risk prediction models may be inadequate.

The 2010 monograph that initially established the concept of CVH identified NHANES data as the most suitable resource for monitoring CVH at the population level (Lloyd‐Jones et al. 2010; Lloyd‐Jones, Ning, et al. 2022). NHANES offers several notable advantages, including its representative sampling from a wide range of demographic groups, the comprehensive inclusion of all cardiovascular health (CVH) metrics—now featuring the newly added sleep metric—across various age categories, as well as in‐person examinations. Additionally, it continues to serve a vital role in population health surveillance conducted by the CDCP. However, NHANES data are not without limitations, including the absence of certain CVH metrics in the youngest age groups (ages under 20 years were excluded in our study).

Niacin (vitamin B3) has garnered attention for its physiological roles and potential cardioprotective properties (Horimatsu et al. 2020; Jia et al. 2020). As a precursor for NAD/NADP coenzymes, niacin is crucial in metabolic pathways like energy metabolism, DNA repair, and antioxidant processes (Biţă et al. 2023; Makarov et al. 2019). Moreover, niacin exerts lipid‐modifying effects by influencing lipoprotein metabolism, impacting lipid profiles. Notably, it reduces LDL‐C, triglycerides, and Lp (a), while increasing HDL‐C (Sahebkar et al. 2016). These favorable lipid changes historically positioned niacin for dyslipidemia management and CVD prevention.

Based on cross‐sectional analyses of NHANES data spanning 2005 to 2018, our study identified a statistically significant positive association between dietary niacin intake and overall cardiovascular health as assessed by the Life's Essential 8 (LE8) score. After adjustment for a broad range of demographic, behavioral, and clinical covariates, each 10 mg/day increase in dietary niacin intake was associated with a 0.65‐point increment in LE8 score. Although the absolute change on a 0–100 scale appears modest (< 0.7%), even small shifts in composite cardiovascular risk scores have been shown in prior literature to correspond with measurable differences in clinical outcomes at the population level. For example, a 1‐point increase in cardiovascular health metrics has been linked with reduced incidence of major adverse cardiovascular events over time (Guo and Zhang 2017). Therefore, the observed relationship, though quantitatively limited, may carry meaningful public health implications, particularly in the context of scalable, diet‐based interventions. This dose–response association was further supported by restricted cubic spline modeling, which confirmed a linear trend with no evidence of nonlinearity. While these findings suggest a potentially beneficial role of dietary niacin intake in supporting cardiovascular health, residual confounding cannot be fully excluded, and longitudinal or interventional studies are needed to clarify causality.

Differences in study design, niacin dosages, and population characteristics may explain the variation in these findings, genetic differences, and niacin dosages. High‐dose niacin used in clinical trials often exceeds typical dietary intake and can lead to adverse effects, whereas our study focused on dietary niacin intake within a range likely reflective of habitual consumption. Additionally, the health effects of niacin might be modulated by individual factors such as baseline nutritional status, genetic predispositions, and the presence of comorbid conditions, which could explain the variability in outcomes. As demonstrated by Asleh et al. (2016), Niacin treatment resulted in a marked improvement in HDL antioxidant function among diabetic patients with the genotype Hp 1–1, while those with the Hp 2–2 experienced deterioration in this function. This suggests that genetic factors may critically influence the response of Niacin, highlighting the need for personalized treatment strategies tailored to the genetic profiles of different populations (Asleh et al. 2016).

Our analysis explored the association between dietary niacin intake and Life's Essential 8 (LE8) cardiovascular health scores, revealing potential effect modification by age, race/ethnicity, socioeconomic status, and cardiovascular disease (CVD) history. As the interaction analyses were not adjusted for multiple comparisons, these findings should be interpreted cautiously as exploratory and hypothesis‐generating rather than conclusive. Nevertheless, the observed patterns are biologically plausible and warrant further investigation.

In particular, we observed notable variation by race/ethnicity and age. Stronger positive associations among non‐Hispanic Whites and non‐Hispanic Blacks may reflect differences in dietary quality, cardiometabolic risk profiles, and genetic factors that influence niacin metabolism and cardiovascular outcomes. The absence of a significant association among Mexican Americans merits further study to identify potential contributors, such as ancestry‐specific metabolic pathways, variations in niacin absorption or utilization, or cultural dietary patterns. Although several studies (Asleh et al. 2016; Hu et al. 2012; Paolini et al. 2023; Safarova and Ezhov 2014; Shin et al. 2012; Tuteja et al. 2017, 2018; Zellner et al. 2005) have evaluated the genetic determinants of response to niacin therapy, there remains a scarcity of population‐level research focused on race‐specific variability in niacin's cardiometabolic effects.

Age‐stratified results further revealed that both younger and older adults appeared to benefit more from higher niacin intake than middle‐aged individuals. This differential response may reflect distinct physiological requirements across life stages. For instance, younger adults may require niacin to support growth and energy metabolism, whereas older adults may experience amplified benefits through niacin's role in modulating lipid profiles, inflammation, and oxidative stress—factors increasingly relevant with advancing cardiovascular risk.

Beyond age and race, our findings suggest that socioeconomic status, as indicated by the poverty‐income ratio (PIR), may influence the relationship between niacin intake and LE8 scores. Participants with higher PIR exhibited more favorable associations, potentially due to greater access to healthful foods, improved dietary diversity, and enhanced nutritional status—all of which can support NAD^+^ biosynthesis and downstream cardioprotective pathways. Conversely, those with lower PIR may rely more heavily on energy‐dense, ultra‐processed foods, which are often poor in micronutrients and can disrupt metabolic homeostasis and NAD^+^ regeneration (Monteiro et al. 2019; Zinöcker and Lindseth 2018). Additionally, chronic socioeconomic disadvantage is linked to persistent systemic inflammation and impaired mitochondrial function, which may further blunt the biological benefits of niacin (Stringhini et al. 2017). Individuals with a history of CVD also demonstrated a stronger association between niacin intake and LE8 scores. This is consistent with known mechanisms, as niacin serves as a precursor for NAD^+^, a coenzyme involved in energy metabolism, redox balance, and vascular repair. Experimental data have shown that NAD^+^‐boosting strategies, including niacin supplementation, can influence key pathways related to lipid metabolism and inflammation (Katsyuba et al. 2020; Trammell et al. 2016). Given that NAD^+^ depletion is a characteristic feature of cardiometabolic disease, nutritional interventions aimed at restoring NAD^+^ levels may hold particular promise for secondary prevention in high‐risk populations (Rajman et al. 2018). Although none of the interaction findings were subjected to formal correction for multiple testing, the patterns identified are biologically coherent and may inform the generation of targeted hypotheses for future studies. These findings underscore the value of considering both biological variability and social context in understanding nutrient–disease relationships and support the advancement of precision nutrition approaches in cardiovascular prevention.

Our study possesses multiple strengths. A large sample that is representative of the nation in NHANES enabled the generalization of our results to the broader US adult population across different racial and age demographics. The comprehensive assessment of LE8 scores provides a holistic measure of cardiovascular health, encompassing health behaviors, factors, and biomarkers across eight domains. To substantially comprehend the relationship between dose and response, we applied restricted cubic splines, which reveal a linear association without evidence of non‐linearity.

Our findings contribute to the evolving understanding of niacin's role in cardiovascular health by highlighting its positive association with LE8 scores in a nationally representative sample. This relationship was especially pronounced among non‐Hispanic Whites, Blacks, and individuals at the younger and older ends of the age spectrum. While previous studies have focused on niacin's effects on lipid profiles or specific CVD outcomes, our study is among the first to evaluate its relationship with the broader, multidimensional LE8 framework introduced by the AHA. These results suggest that promoting adequate dietary niacin intake—through nutrient‐rich foods such as poultry, fish, whole grains, and legumes—could be a practical consideration in future dietary guidance aimed at improving LE8‐related cardiovascular health. However, due to the cross‐sectional design of this study, causality cannot be inferred. Additionally, dietary intake was based on 24‐h recall, which is subject to recall bias and measurement error. These limitations are particularly relevant for LE8, which integrates both self‐reported behavioral factors (e.g., diet, physical activity, smoking) and clinical measures. Consequently, the observed associations may be attenuated or confounded by reporting inaccuracies or unmeasured variables, and should be interpreted with caution. Future prospective studies or randomized dietary interventions are warranted to validate these findings.

## 5 Conclusions

In this cross‐sectional analysis, higher dietary niacin intake was positively associated with better cardiovascular health status, as reflected by the Life's Essential 8 (LE8) score. Exploratory subgroup analyses suggested that the strength of this association may differ across demographic and clinical characteristics, with notable variations observed by race/ethnicity, age, socioeconomic status, and cardiovascular disease history. While these patterns may offer valuable insights for hypothesis generation and potential directions for tailored dietary guidance, they should be interpreted cautiously given the observational design and the absence of formal interaction testing for causality. Further longitudinal and interventional studies are needed to confirm these associations, examine potential effect modification, and clarify the role of dietary niacin within multidimensional frameworks of cardiovascular health promotion.

## Author Contributions

**Dan Wang:** conceptualization (equal), formal analysis (equal), funding acquisition (equal), investigation (equal), resources (equal), writing – original draft (equal), writing – review and editing (equal). **Fanli Yuan:** conceptualization (equal), formal analysis (equal), investigation (equal), resources (equal), writing – original draft (equal), writing – review and editing (equal). **Shouliang Hu:** conceptualization (equal), data curation (lead), formal analysis (equal), funding acquisition (equal), methodology (lead), project administration (lead), supervision (lead), writing – original draft (equal), writing – review and editing (equal).

## Ethics Statement

Approval for the NHANES was granted by the National Center for Health Statistics Research Ethics Review Board.

## Conflicts of Interest

The authors declare no conflicts of interest.

## Supporting information


**Table S1:** Life's Essential 8 (LE8) component scoring criteria.
**Table S2:** Healthy Eating Index‐2015 (HEI‐2015) components and scoring method.
**Table S3:** Univariate analyses of LE8 scores by participant characteristics.
**Table S4:** Associations between dietary niacin intake and LE8 subdomain scores.
**Table S5:** Sensitivity analysis using niacin intake from two 24‐hour recalls.

## Data Availability

The data utilized in this research were sourced from the National Health and Nutrition Examination Survey (NHANES) covering the period from 2005 to 2018, which is publicly accessible online: https://wwwn.cdc.gov/nchs/nhanes/Default.aspx.
