# Supplementary material for: Association Between Dietary Niacin Intake and Life's Essential 8 Among US Adults (NHANES 2005–2018)
Source: Food Sci Nutr. 2025 Sep 2;13(9):e70817. doi: 10.1002/fsn3.70817 (PMC12402905; doi:10.1002/fsn3.70817)
Supplement: Supplementary file 1 — Table S1: Life's Essential 8 (LE8) component scoring criteria. Table S2: Healthy Eating Index‐2015 (HEI‐2015) components and scoring method. Table S3: Univariate analyses of LE8 scores by participant characteristics. Table S4: Associations between dietary niacin intake and LE8 subdomain scores. Table S5: Sensitivity analysis using niacin intake from two 24‐hour recalls. [file FSN3-13-e70817-s001.docx]

**Supplemental Online Content**

**Supplemental TABLE S1 |** Life’s Essential 8 (LE8) Component Scoring Criteria.

**Supplemental TABLE S2 |** Healthy Eating Index-2015 (HEI-2015) Components and Scoring Method.

**Supplemental TABLE S3 |** Univariate analyses of LE8 scores by Participant Characteristics.

**Supplemental TABLE S4 |** Associations between Dietary Niacin Intake and LE8 Subdomain Scores.

**Supplemental TABLE S5 |** Sensitivity Analysis Using Niacin Intake from Two 24-Hour Recalls.

**Supplemental TABLE S1 |** Life’s Essential 8 (LE8) Component Scoring Criteria.

|  |  |  |  | Quantification of CVH Metric | | |
| --- | --- | --- | --- | --- | --- | --- |
| Health Metric |  | Method of Measurement |  | Points |  | Measured Value |
| **Behavioral Domain** | | | | | | |
| Dietary  Intake |  | Calculated Healthy Eating Index (HEI)  2015 derived from self-reported 24-hr  recall via the internet-based Automated  Self-Administered Dietary Assessment  Tool (ASA24) |  | 100 |  | ≥95th percentile |
|  |  |  |  | 80 |  | 75th - 94th percentile |
|  |  |  |  | 50 |  | 50th - 74th percentile |
|  |  |  |  | 25 |  | 25th - 49th percentile |
|  |  |  |  | 0 |  | <25th percentile |
| Physical  Activity |  | Self-reported minutes/week of  moderate or higher intensity activity |  | 100 |  | ≥150 minutes |
|  |  |  |  | 90 |  | 120 - 149 minutes |
|  |  |  |  | 80 |  | 90 - 119 minutes |
|  |  |  |  | 60 |  | 60 - 89 minutes |
|  |  |  |  | 40 |  | 30 - 59 minutes |
|  |  |  |  | 20 |  | 1 - 29 minutes |
|  |  |  |  | 0 |  | 0 minutes |
| Smoking  Status |  | Self-reported history of cigarette use,  inhaled nicotine delivery system (e- cigarettes, vaping devices) use, or  secondhand smoke exposure at home |  | 100 |  | Never |
|  |  |  |  | 50 |  | Previous |
|  |  |  |  | 0 |  | Current |
|  |  |  |  | -20 |  | Subtract 20 points if living with indoor smoker^a^ |
| Sleep  Health |  | Weighted average of self-reported  hours of sleep/night for the prior month  reported separately for week and  weekend days |  | 100 |  | ≥7 to <9 h/day |
|  |  |  |  | 90 |  | ≥9 to <10 h/day |
|  |  |  |  | 70 |  | ≥6 to <7 h/day |
|  |  |  |  | 40 |  | ≥5 to <6 or ≥10 h/day |
|  |  |  |  | 20 |  | ≥4 to <5 h/day |
|  |  |  |  | 0 |  | <4 h/day |
| **Biomedical Domain** | | | | | | |
| BMI |  | Weight (kg) measured with Tanita  electronic scale (model TBF-3001,  Arlington Heights, IL) |  | 100 |  | <25 kg/m2 |
|  |  |  |  | 70 |  | 25.0 to 29.9 kg/m2 |
|  |  |  |  | 30 |  | 30.0 to 34.9 kg/m2 |
|  |  | Height (m) measured with portable  standing stadiometer (model SECA 213,  Seca Worldwide, Hamburg, Germany) |  | 15 |  | 35.0 to 39.9 kg/m2 |
|  |  |  |  | 0 |  | ≥40.0 kg/m2 |
| Blood  Pressure |  | Average of five blood pressure  measurements with the OMRON  Professional IntelliSense digital blood  pressure monitor (Model HEM-907XL,  OMRON Healthcare, Inc., Kyoto, Japan) |  | 100 |  | <120 & <80 mmHg |
|  |  |  |  | 75 |  | ≥120 to 129 & <80 mmHg |
|  |  |  |  | 50 |  | ≥130 to 139 or ≥80 to 89 mmHg |
|  |  |  |  | 25 |  | ≥140 to 159 or ≥90 to 99 mmHg |
|  |  |  |  | 0 |  | ≥160 or ≥100mmHG |
|  |  |  |  | -20 |  | Subtract 20 points if medication treated^a^ |
| Blood  Lipids^b^ |  | Non-HDL cholesterol calculated as  difference between plasma total  cholesterol and HDL-cholesterol (mg/dL) |  | 100 |  | <130 mg/dL |
|  |  |  |  | 60 |  | ≥130 to 189 mg/dL |
|  |  |  |  | 40 |  | ≥160-189 mg/dL |
|  |  |  |  | 20 |  | ≥190 to 219 mg/dL |
|  |  |  |  | 0 |  | ≥220 mg/dL |
|  |  |  |  | -20 |  | Subtract 20 points if medication treated^a^ |
| Glycemia^b^ |  | Fasting blood glucose (FBG, mg/dL) or  casual hemoglobin A1c (%), plus self-reported diabetes mellitus at the midlife visit |  | 100 |  | No history of diabetes + FBG <100 or HbA1c <5.7% |
|  |  |  |  | 60 |  | No history of diabetes + FBG 100-125 or HbA1c 5.7-6.4% |
|  |  |  |  | 40 |  | Diabetes + HbA1c <7.0% |
|  |  |  |  | 30 |  | Diabetes + HbA1c 7.0 - 7.9% |
|  |  |  |  | 20 |  | Diabetes + HbA1c 8.0 - 8.9% |
|  |  |  |  | 10 |  | Diabetes + HbA1c 9.0 - 9.9% |
|  |  |  |  | 0 |  | Diabetes + HbA1c ≥10.0.% |

All metrics scored from 0 to 100, with a higher score indicating a healthier score.

Overall LE8 score is an average of all eight component scores.

^a^ Only subtract points if score is not 0 points;

^b^ Components of the Blood Subdomain.

**Supplemental TABLE S2 |** Healthy Eating Index-2015 (HEI-2015) Components and Scoring Method.

| **Component** |  | **Maximum points** |  | **Standard for maximum score** |  | **Standard for minimum score of zero** |
| --- | --- | --- | --- | --- | --- | --- |
| **Adequacy** | | | | | | |
| Total Fruits^b^ |  | 5 |  | ≥0.8 cup equiv. per 1,000 kcal |  | No Fruits |
| Whole Fruits^c^ |  | 5 |  | ≥0.4 cup equiv. per 1,000 kcal |  | No Whole Fruits |
| Total Vegetables^d^ |  | 5 |  | ≥1.1 cup equiv. per 1,000 kcal |  | No Vegetables |
| Greens and Beans^d^ |  | 5 |  | ≥0.2 cup equiv. per 1,000 kcal |  | No Dark Green Vegetables or Legumes |
| Whole Grains |  | 10 |  | ≥1.5 oz equiv. per 1,000 kcal |  | No Whole Grains |
| Dairy^e^ |  | 10 |  | ≥1.3 cup equiv. per 1,000 kcal |  | No Dairy |
| Total Protein Foods^f^ |  | 5 |  | ≥2.5 oz equiv. per 1,000 kcal |  | No Protein Foods |
| Seafood and Plant Proteins^f, g^ |  | 5 |  | ≥0.8 oz equiv. per 1,000 kcal |  | No Seafood or Plant Proteins |
| Fatty Acids^g^ |  | 10 |  | (PUFAs + MUFAs)/SFAs ≥2.5 |  | (PUFAs + MUFAs)/SFAs ≤1.2 |
| **Moderation** |  |  |  |  |  |  |
| Refined Grains |  | 10 |  | ≤1.8 oz equiv. per 1,000 kcal |  | ≥4.3 oz equiv. per 1,000 kcal |
| Sodium |  | 10 |  | ≤1.1 gram per 1,000 kcal |  | ≥2.0 grams per 1,000 kcal |
| Added Sugars |  | 10 |  | ≤6.5% of energy |  | ≥26% of energy |
| Saturated Fats |  | 10 |  | ≤8% of energy |  | ≥16% of energy |

^a^ Intakes between the minimum and maximum standards are scored proportionately.

^b^ Includes 100% fruit juice.

^c^ Includes all forms except juice.

^d^ Includes legumes (beans and peas).

^e^ Includes all milk products, such as fluid milk, yogurt, and cheese, and fortified soy beverages.

^f^ Includes seafood, nuts, seeds, soy products (other than beverages), and beans, peas, and lentils.

^g^ Ratio of poly- and monounsaturated fatty acids (PUFAs and MUFAs) to saturated fatty acids (SFAs).

**Supplemental TABLE S3 |** Univariate analyses of LE8 scores by Participant Characteristics.

| Item | β (95%CI) | *P* |
| --- | --- | --- |
| Niacin_Per10 | **0.83 (0.69, 0.96)** | **< 0.001** |
| Niacin_Per10 group | | |
| <17.7 mg/d  17.7-27.1 mg/d | ref^a^  **1.67 (1.21, 2.13)** | ref^a^  **< 0.001** |
| ≥27.1 mg/d | **3.12 (2.66, 3.58)** | **< 0.001** |
| Age | **-0.26 (-0.27, -0.25)** | **< 0.001** |
| Age group | | |
| <40  40-65 | ref^a^  **-8.8 (-9.21, -8.38)** | ref^a^  **< 0.001** |
| ≥65 | **-10.78 (-11.27, -10.3)** | **< 0.001** |
| Sex (female vs male) | **1.92 (1.54, 2.3)** | **< 0.001** |
| Race | | |
| Mexican American  Non-Hispanic black | ref^a^  **-3.81 (-4.45, -3.16)** | ref^a^  **< 0.001** |
| Non-Hispanic white | **1.08 (0.52, 1.63)** | **< 0.001** |
| Others | **3.36 (2.72, 4.01)** | **< 0.001** |
| PIR | **1.91 (1.8, 2.02)** | **< 0.001** |
| PIR_group | | |
| <1.0  1.0-3.0 | ref^a^  **1.47 (0.96, 1.98)** | ref^a^  **< 0.001** |
| ≥3.0 | **6.86 (6.35, 7.38)** | **< 0.001** |
| Education | | |
| Less than high school  High school or equivalent | ref^a^  **2.41 (1.86, 2.95)** | ref^a^  **< 0.001** |
| Above high school | **8.58 (8.12, 9.04)** | **< 0.001** |
| Marital (Married vs Living alone) | **-1.62 (-2.01, -1.24)** | **< 0.001** |
| drinking status | | |
| Never  Former | ref^a^  **-6.42 (-7, -5.84)** | ref^a^  **< 0.001** |
| Current | **0.59 (0.16, 1.02)** | **0.007** |
| Depression (yes vs no) | **-8.93 (-9.63, -8.23)** | **< 0.001** |
| CVD (yes vs no) | **-9.58 (-10.18, -8.98)** | **< 0.001** |
| CKD (yes vs no) | **-8.93 (-9.4, -8.45)** | **< 0.001** |
| Energy | **0.0009 (0.0006, 0.0011)** | **< 0.001** |
| Antihypertension drugs (yes vs no) | **-7.62 (-8.09, -7.14)** | **< 0.001** |
| lipid-lowering drugs (yes vs no) | **-10.72 (-11.11, -10.34)** | **< 0.001** |

^a^ref, reference.

**Supplemental TABLE S4 |** Associations between Dietary Niacin Intake and LE8 Subdomain Scores.

| **Outcomes** |  | **β (95% CI)** | | | | |
| --- | --- | --- | --- | --- | --- | --- |
|  |  | **Model 1** |  | **Model 2** |  | **Model 3** |
| LE8 overall score |  | **0.77 (0.56, 0.97)** |  | **0.62 (0.42, 0.82)** |  | **0.65 (0.43, 0.87)** |
| LE8 Behavioral domain^a^ |  | **0.90 (0.60, 1.20)** |  | **1.02 (0.69, 1.36)** |  | **1.01 (0.69, 1.33)** |
| LE8 Biomedical domain^b^ |  | **0.63 (0.35, 0.92)** |  | 0.21 (-0.04, 0.46) |  | 0.28 (-0.01, 0.58) |
| LE8 Blood biomarkers subdomain^c^ |  | **0.88 (0.57, 1.19)** |  | **0.48 (0.19, 0.77)** |  | **0.44 (0.10, 0.77)** |

^a^ Behavioral domain includes diet, physical activity, smoking status, and sleep duration.

^b^ Biomedical domain includes body mass index, blood pressure, blood lipids (non–high-density lipoprotein cholesterol), and glycemia.

^c^ Blood biomarkers subdomain includes blood lipids and glycemia.

Model1: Crude.

Model2: Age/Sex/Race-Adjusted.

Model3: Fully Adjusted.

**Supplemental TABLE S5 |** Sensitivity Analysis Using Niacin Intake from Two 24-Hour Recalls.

| **Variable** | **β (95% CI)** | | | | | | | | | |
| --- | --- | --- | --- | --- | --- | --- | --- | --- | --- | --- |
|  | **No.** | **Model 1** |  | ***p***-Value | **Model 2** |  | ***p***-Value | **Model 3** |  | ***p***-Value |
| Dietary niacin intake | 21,030 | 0.91 (0.66, 1.16) |  | <0.001 | 0.72 (0.48, 0.97) |  | <0.001 | **0.62 (0.13, 0.58)** |  | **<0.001** |
| **Tertiles** |  |  |  |  |  |  |  |  |  |  |
| Q1(<18.6mg/d) | 7010 | 1(Ref) |  |  | 1(Ref) |  |  | 1(Ref) |  |  |
| Q2(18.6-28.9mg/d) | 7010 | 1.75 (0.89, 2.61) |  | <0.001 | 1.67 (0.87, 2.48) |  | <0.001 | **1.01 (0.31, 1.71)** |  | **0.005** |
| Q3(≥28.9mg/d) | 7010 | 2.67 (1.95, 3.40) |  | <0.001 | 2.36 (1.59, 3.14) |  | <0.001 | **1.72 (0.93, 2.50)** |  | **<0.001** |
| *P* for trend |  |  |  | <0.001 |  |  | <0.001 |  |  | **<0.001** |

Model1: Crude.

Model2: Age/Sex/Race-Adjusted.

Model3: Fully Adjusted.
